# The Constructive Nature of Affective Vision: Seeing Fearful Scenes Activates Extrastriate Body Area

**DOI:** 10.1371/journal.pone.0038118

**Published:** 2012-06-29

**Authors:** Charlotte B. A. Sinke, Jan Van den Stock, Rainer Goebel, Beatrice de Gelder

**Affiliations:** 1 Cognitive and Affective Neurosciences Laboratory, Tilburg University, Tilburg, The Netherlands; 2 Faculty of Psychology and Neuroscience, Maastricht University, Maastricht, The Netherlands; 3 Martinos Center for Biomedical Imaging, Massachusetts General Hospital, Harvard Medical School, Charlestown, Massachusetts, United States of America; French National Centre for Scientific Research, France

## Abstract

It is part of basic emotions like fear or anger that they prepare the brain to act adaptively. Hence scenes representing emotional events are normally associated with characteristic adaptive behavior. Normally, face and body representation areas in the brain are modulated by these emotions when presented in the face or body. Here, we provide neuroimaging evidence (using functional magnetic resonance imaging) that the extrastriate body area (EBA) is highly responsive when subjects observe isolated faces presented in emotional scenes. This response of EBA to threatening scenes in which no body is present gives rise to speculation about its function. We discuss the possibility that the brain reacts proactively to the emotional meaning of the scene.

## Introduction

There is increasing evidence that the perceptual system is constructive and actively fills in and anticipates information rather than passively representing given stimuli [1], [2]. Typical contexts can trigger the representation of an object which is not physically present in the scene the observer is watching [3]. For example, when viewing a scene in which the occurrence of faces is highly probable, the fusiform face area (FFA), a brain area normally responsive to seen faces, is active even though not a single face is shown in the scenes [4]. These constructive abilities of perception appear to be especially useful in case of affective stimuli [5]. Indeed, since Darwin, it has been argued that preparing the organism for future adaptive action is at the core of emotion states. In line with this, visual scenes representing highly emotional events are associated in our mind with the appropriate actions [6]. For instance, when viewing an image of an explosion or of a house on fire, it is part of our understanding of the affective significance of the image to complete the picture by imagining people running away.

This paper reports findings that are consistent with this notion. The results presented here are part of a larger study using functional magnetic resonance imaging (fMRI) and designed to investigate the influence of affective pictures on processing facial expressions. Specifically, we wanted to know whether the fearful emotion triggered by the scene would increase activation in face processing areas. In a previous study, it did heighten the amplitude of the N170, an event-related potential related to the processing of faces, for both neutral and fearful faces in a fearful vs. a neutral context [7]. For this study, we used neutral and fearful scene stimuli with a neutral or fearful facial expression overlaid on them, as well as controls for the scenes and faces separately. Our design did not focus on bodily expressions and we did not have predictions about body processing areas. Therefore we choose to report and discuss this current finding about the seemingly significant role of extrastriate body area (EBA) separately.

The EBA is an area in the lateral occipitotemporal cortex which is highly responsive to observing bodies and even more so when the body shows a dynamic emotion [8], [9]. Although the stimuli in this study did not show bodies, we found this area activated. To investigate this, we compared the scene-only and the scenes-faces conditions to rule out that the putative activity in EBA was simply due to perceptual stimulus completion. This might have happened since in all the stimuli containing faces, the same geometrical figure was positioned below the face in order to wipe out the impression of free floating faces. In line with emotional action readiness theory we conjectured that this body related brain activity could also reveal the specific valence of the scene and reflect the viewer’s automatic associations with it. An action readiness perspective on emotions holds that different emotions lead to different states of action tendencies in the observer, either to approach or move away from the emotional source [10]. Following up on this theory, activation of body related areas in the brain for concealed bodies might be highest when subjects view a fear evoking scene. This may than be taken as an indication that the brain anticipates the bodily action appropriate for the scene. Bodies are known to specifically activate EBA [11] and fusiform body area (FBA [12]). In contrast to FBA, EBA is spatially separated from the FFA so there can be no confusion about its possible activation related to the non-present bodies.

## Materials and Methods

### Participants

Fifteen healthy volunteers (six male; 26.2±5.9 years; all right-handed) participated in this experiment, but one subject was excluded from analysis due to excessive head movement. The study was performed in accordance to the *Declaration of Helsinki* and was approved by the Ethics Committee of the faculty of Psychology and Neuroscience (ECP Maastricht, the Netherlands). All participants gave written informed consent.

### Design

**Figure 1:**
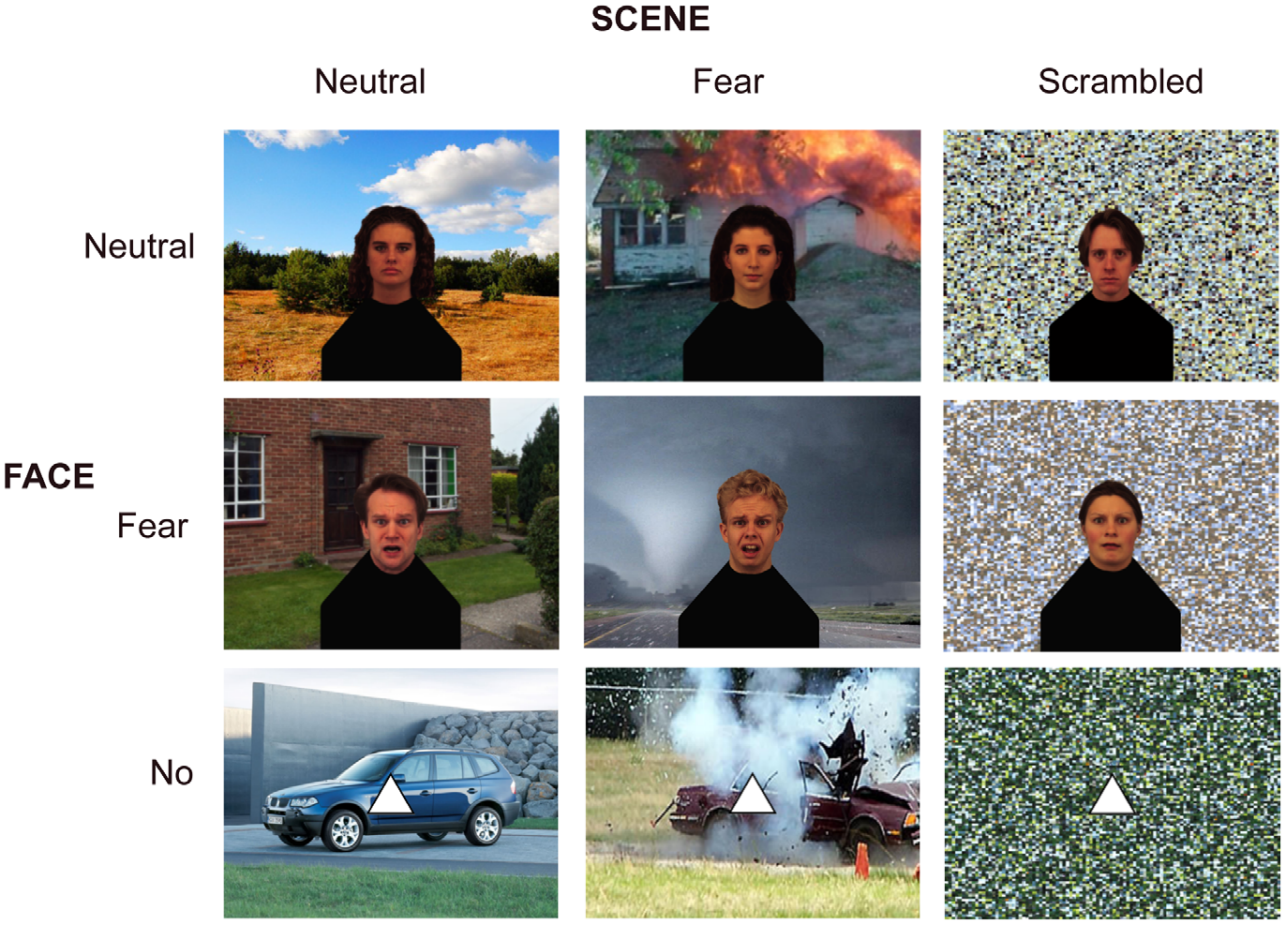
3×3 factorial design. Neutral and fearful faces were overlaid centrally on a neutral or fearful scene. As controls, scrambled scenes and triangles instead of faces were used.

We created nine stimulus conditions leading to a 3×3 design (see Fig. 1). A neutral (Nf) or a fearful face (Ff) was placed in the middle of a neutral (Ns) or a fearful scene (Fs). As control conditions, a face could also appear on scrambled scene background (Xs), and instead of a face a triangle (Xf) could appear on top of the three background types. Underneath all faces in all conditions, the same body-like shape was placed so no specific information could be extracted from those. 24 different scenes (half neutral, half fearful) and 24 different faces (half male; half neutral, half fearful) from the Karolinska Directed Emotional Face database [13] were used. Each identity was used in all conditions.

A blocked functional magnetic resonance imaging (fMRI) design was used. In one stimulus block of 9 s, eight stimuli were presented for 800 ms with an inter stimulus interval (only fixation cross) of 325 s. Subjects had to press a button whenever an oddball (an inverted picture) appeared. The aim of this task was to keep participant’s attention on the screen. Blocks including an inverted picture were discarded from analysis. Fixation blocks separated stimulus blocks with a duration of 15.75 s. In total, 108 stimulus blocks (excluding sixteen interleaved oddball blocks) were presented in four runs.

An independent localizer run was used to locate face processing areas in each individual. This localizer is frequently used for different studies in our lab. Since it also contains blocks of bodies, this later gave us the opportunity to also locate EBA per subject after this area caught our attention. This run comprised 20 stimulation blocks of 12 s, interleaved with 14 s fixation blocks. Stimulation blocks contained twelve pictures of either bodies, faces (different ones than those used in the main experiment), houses or tools, each presented for 450 ms with an inter stimulus interval of 600 ms. Here, a one-back task was used. Total run duration was 8 m 54 s.

### Data Acquisition

Scanning was performed in a 3T head scanner (Siemens Allegra, AG, Erlangen, Germany) using a standard quadrature birdcage head coil. For the experimental scan, the following scan parameters were used: TR = 2250 ms; TE = 25 ms; 42 slices of 2.5 mm (no gap); leading to a resolution of 3.5×3.5×2.5 mm. For the localizer scan, different parameters were used to achieve a higher resolution of 2×2×2 mm: TR = 2000 ms; TE = 30 ms; 28 slices of 2 mm (no gap).

### Data Analysis

For the fMRI data analysis BrainVoyager QX (version 1.10.4, Brain Innovation, Maastricht, the Netherlands) was used. Before statistical data analysis, data were cleared for scanner-related signal drifts and head movements, temporally high-pass filtered, transformed into Talairach (Tal) space and spatially smoothed with a 4 mm Gaussian kernel. The first two scans per run were excluded from the analysis to permit T1 equilibration effects.

For the whole brain analysis, a multi-subject general linear model (GLM) was run using a regression model consisting of the nine predictors corresponding to the experimental conditions plus one for the oddball blocks. The predictor time courses used were generated on the basis of a linear model of the relation between neural activation and hemodynamic response. For our main study, a whole brain random effects ANOVA with two within-participants factors (face, scene) with three levels (neutral, fearful, scramble/triangle) was performed. Investigating these data, our attention was caught when we looked at the contrast FfFs>NfNs, showing an area that we recognized as EBA. To test whether this was indeed EBA, we performed two checks. First, we functionally localized the EBA on group level with the localizer data to see whether it overlapped with the cluster. Secondly, the cluster found with contrast FfFs>NfNs was subjected to a paired-samples t-test with the group localizer data to make sure it was indeed body selective.

**Figure 2:**
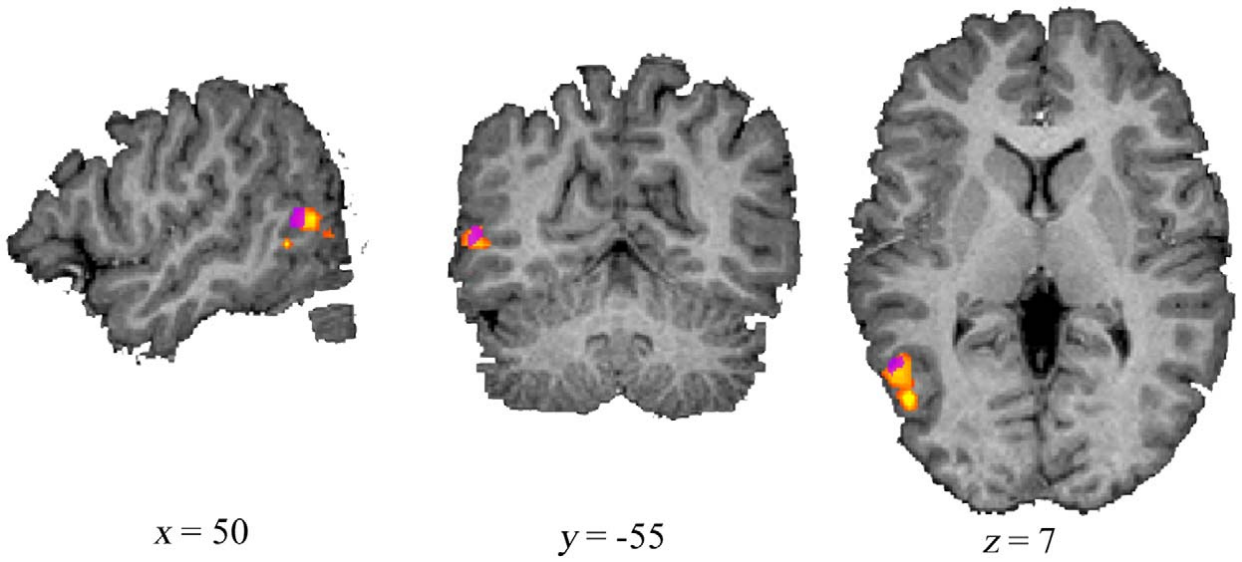
Overlap between experimentally found and functionally localized EBA. EBA was experimentally found with contrast Fearful face in Fearful scene > Neutral face in Neutral scene on whole brain level (*p*<.005; purple cluster). The group activation was found for bodies > faces + tools + houses with the functional localizer (FDR (*q*)<.003; yellow/orange cluster).

Since both checks showed the body selectivity of the region, we continued with using the localizer scan to define right EBA independently per subject as region-of-interest (ROI) to be able to perform a more specialized analysis. Also, we localized FFA (faces > houses & tools), which will be discussed here to show the specificity of the effect. EBA was located with at least a False Discovery Rate (FDR) correction of *q*<.01; only in 2 subjects a more liberal threshold of *p*<.02 was used due to otherwise small cluster sizes. FFA ROIs were chosen with at least FDR(*q*)<.1 and only 1 subject at *p*<.02, to be able to get cluster sizes of 50–200 voxels. From the individually located EBAs, a subject-specific ROI-based group ANOVA (two within-participants factors (face, scene) with three levels (neutral, fearful, scramble/triangle)) was performed with the experimental data, followed by various paired-samples t-tests using SPSS (Version 15.0).

## Results

Our comparison of fearful faces within a fearful scene versus neutral faces within a neutral scene at the whole brain level, revealed an area within right lateral occipito-temporal cortex. When comparing this to the body specific activation found with the separate functional localizer scan on group level, they indeed showed overlap (see Fig. 2). Similarly, comparing fearful vs. neutral scenes without faces revealed the same area. The fact that these simple effects are found at the whole brain level shows the robustness of the following ROI findings.

**Figure 3:**
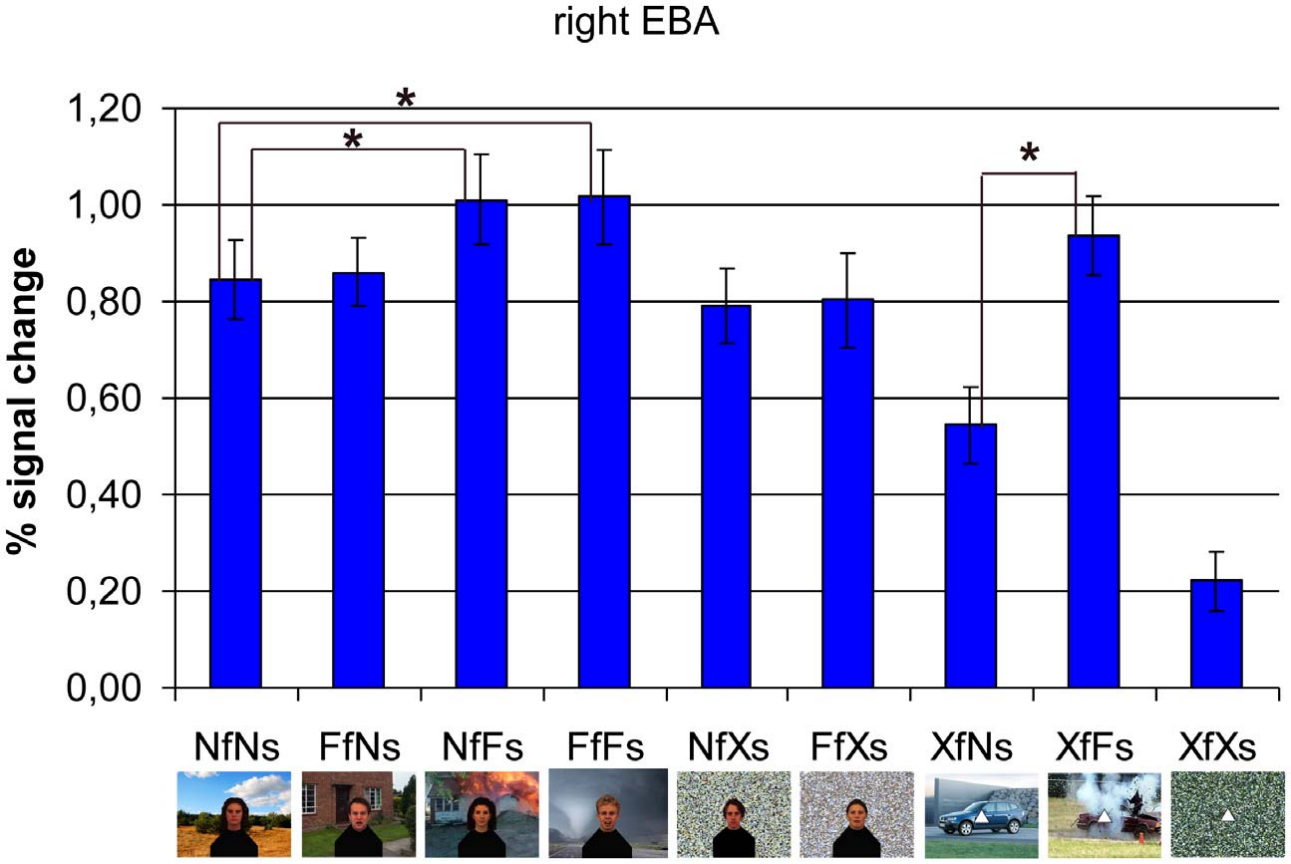
Subject-specific region-of-interest (ROI)-based group analysis in right extrastriate body area (EBA). There is an interaction between facial and scene emotion. The region shows more activation for fear presented in both contexts than to no emotion at all (FfFs >NfNs: *p*<.007). This effect is probably caused by the emotion from the scene (NfFs > NfNs: *p*<.011) and not by emotion from the face (FfNs > NfNs: n.s.), especially since threatening scenes without face also activate EBA (XfFs >XfNs: *p*<.000). N = neutral; F = fearful; f = face; s = scene; X = control (scrambled scene or no face).

The additional paired-samples t-test with the localizer data in the whole brain FfFs>NfNs cluster showed that bodies gave rise to more activation in this region than faces (*t*(13) = 6.130, *p = *.000, *d* = 1.079), houses (*t*(13) = 8.830, *p = *.000, *d* = 1.758) and tools (*t*(13) = 7.298, *p = *.000, *d* = 1.303). There was no difference in activation between houses, faces and tools. This result illustrates the strong body selectivity of this cluster.

In all participants it was possible to locate EBA in the right hemisphere, and all individual Tal coordinates fell within the range of those reported in different studies as investigated in our review [14] (see Table S1 and Fig. S1).

The subject-specific ROI-based group ANOVA showed an interaction between facial and scenery emotion (*F*(4,10) = 11.309, *p*<.001, η_p2_ = .819). Paired-samples t-tests showed that fearful faces in fearful scenes gave rise to higher activation than neutral faces in neutral scenes (FfFs > NfNs; *t*(13) = 3.207, *p = *.007, *d* = .259). This contextual emotion effect in EBA seemed to be caused by threat from the scenes (NfFs > NfNs; *t*(13) = 2.958, *p = *.011, *d* = .257), not by fear from the faces (FfNs > NfNs; *t*(13) = 0.281, *p = *n.s., *d* = .029). This is especially clear when looking at the stimuli without faces which produced the strongest effect; threatening scenes gave rise to higher EBA activity than neutral scenes (XfFs > XfNs; *t*(13) = 5.814, *p = *.000, *d* = .658). Also, there was a trend for more activation for fearful faces when they appeared in a fearful scene (FfFs > FfNs; *t*(13) = 2.020, *p = *.064, *d* = .248). See Figure 3 for the average hemodynamic responses within right EBA per condition.

To see whether those emotional scene effects are specific to EBA, we performed the same subject-specific ROI-based group analysis in right FFA (see Fig. S2). Here we also found an interaction (*F*(4,10) = 11.258, *p*<.001, η_p2_ = .818). However, in contrast with EBA, this was not due to higher activation specifically in case of a threatening scene. Only when there were no faces, this area responded more to fearful than neutral scenes (XfFs > XfNs; *t*(13) = 3.638, *p = *.003, *d* = .491), but this activation was still much lower than the activation for scenes including faces (whether being emotional or not). So adding a fearful face to the fearful scene increased the response in right FFA (FfFs > XfFs; *t*(13) = 5.072, *p = *.000, *d* = 1.732).

## Discussion

Our results show that EBA can get activated solely by a threatening scene in which there is no body present. This indicates that the constructive processes of the brain go beyond merely activating the representation of a stimulus not explicitly represented. The EBA activation was specifically associated with threatening scenes since it was also observed for threatening scenes when there was no face present, and was as high as when faces were included. Although mental imagery can activate the corresponding object category in the brain [15], we believe the activation found here is not simply due to imagination of the body since FFA reacted significantly less to the no-face stimuli. Also in the study of Cox and colleagues [4], FFA responded to blurred faces when presented on top of a body, but not when the blurred faces were presented in isolation even though it should have been clear to the subjects that it were faces since it obviously was seen so in the other experimental conditions. Furthermore, there was a significant difference in EBA between scene stimuli with and without faces. So, the fact that we do find EBA specifically for threatening scenes without faces present may suggest that the observed EBA activation reflects associative and anticipatory capabilities of the brain. This interpretation is quite speculative, but in line with this, previous studies have shown that the brain is able to do this very quickly. Orbitofrontal cortex seems to make predictions of possible representations even before the stimulus is recognized in the corresponding visual object processing areas [16]. These predictions are based on memory of past experiences, mental simulation, imagery, and contextual cues. Some even argue that the brain is actually continuously generating predictions [17]. Also, anticipation of a stimulus has been shown to activate the same regions that are found active for the actual sensory input [18].

An alternative explanation for the observed EBA activation needs to be explored in future work. The observed EBA activity could be related to the participant’s own bodily awareness triggered by the fear scene and reflect the observer’s body posture in such a case. In that case, one expects that EBA activation would also be found in other studies presenting threatening stimuli even though no bodies are presented. But unfortunately, in many emotion studies was only looked in specific ROIs like AMG or FFA and the question raised here can not be verified post hoc. Even so, when no EBA modulation is found as in a recent study whereby subjects viewed hands either in pain or not [19], it may also be the case that the stimuli do not present a real immediate threat for the observer such as to induce a response in this area.

Interestingly, regions that are involved in body schema and action awareness representations are in close proximity to EBA, like the angular gyrus [20], [21]. And EBA itself, in addition to visual processing, also appears to integrate sensory-motor signals related to the representation of your own body, also when no real motion is involved as is the case during motor imagery [22]. In a very recent study, Kühn and colleagues found EBA activated together with hand-related areas of the motor cortex when subjects were anticipating having to make a hand movement [23]. This again suggests that EBA plays a role in representing the own body. Finally, it seems to be involved in a network, together with right temporoparietal junction and posterior superior temporal gyrus, activated during out-of-body experiences [24].

There was no condition specific effect in EBA for emotional faces. A possible reason for this may be that attention was on the whole stimulus and as the scene covers more space than the face it may have absorbed the most attention. However, we suggest that it is more plausible that a threatening scene provides more cues about bodily behavior and action than provided by an isolated fearful face. Future studies could include heart and breathing rate measurements to measure bodily responses to the threatening scene.

In conclusion, our findings possibly provide neural evidence for the role of emotional contextual cues and may be taken to suggest that the brain reacts to the meaning of the scene by projecting the bodily behavior associated with the scene. This result was obtained by pursuing a different question addressed in a fuller design, but we believe it is worth reporting for its own sake. Indeed, the present result illustrates for the first time for the field of affective perception the constructive properties of the visual system which have been highlighted already for scene and object perception.

## Supporting Information

Figure S1 **All individually localized right EBA clusters.**
(TIF)

Figure S2 **Subject-specific region-of-interest (ROI) group analysis in right fusiform face area (FFA).** Those were individually localized by an independent localizer run. There is an interaction between facial and scenery emotion. Adding a fearful face to a fearful scene increases activation (FfFs > XfFs: *p*<.000). Fearful vs. neutral scenes without faces shows higher activation in right FFA (XfFs > XfNs: *p*<.003). N = neutral; F = fearful; f = face; s = scene; X = control (scrambled scene or triangle).
(TIF)

Table S1 **All individual Tal coordinates per subject.**
(DOC)
