# Supplementary figures and images for: The Constructive Nature of Affective Vision: Seeing Fearful Scenes Activates Extrastriate Body Area
Source: PLoS One. 2012 Jun 29;7(6):e38118. doi: 10.1371/journal.pone.0038118 (PMC3387182; doi:10.1371/journal.pone.0038118)

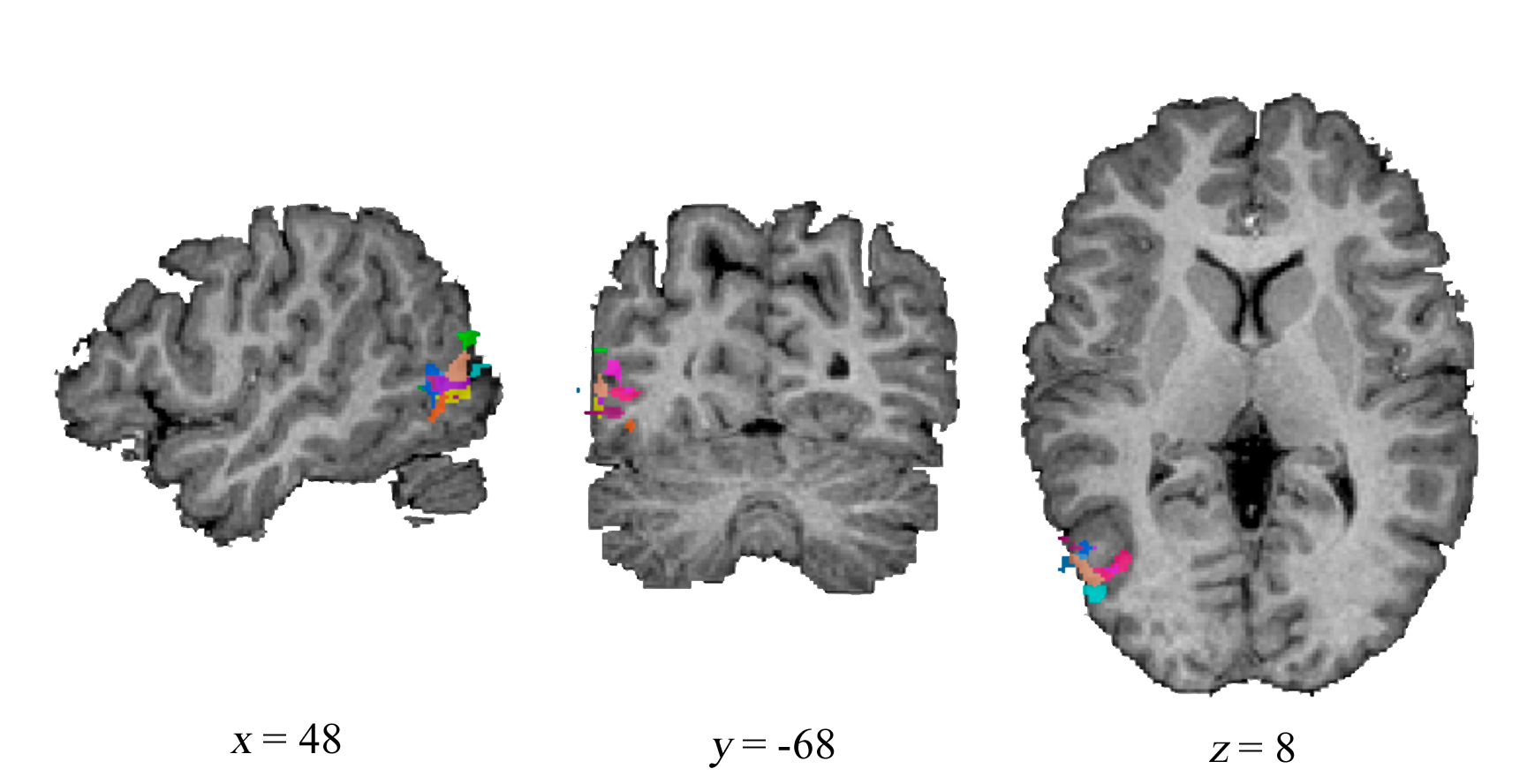

Supplement: Figure S1 — All individually localized right EBA clusters. (TIF) [file pone.0038118.s001.tif]

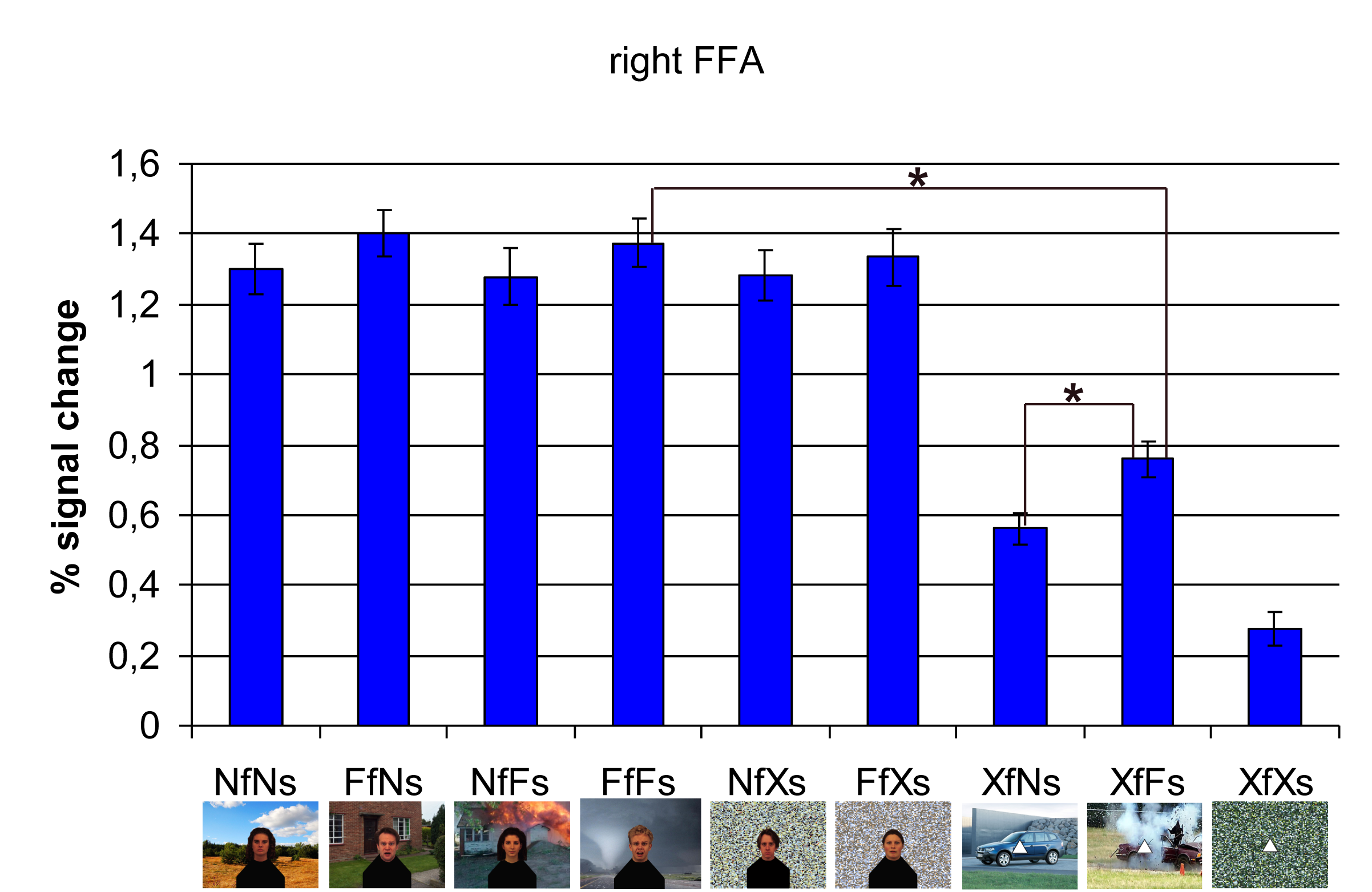

Supplement: Figure S2 — Subject-specific region-of-interest (ROI) group analysis in right fusiform face area (FFA). Those were individually localized by an independent localizer run. There is an interaction between facial and scenery emotion. Adding a fearful face to a fearful scene increases activation (FfFs > XfFs: p<.000). Fearful vs. neutral scenes without faces shows higher activation in right FFA (XfFs > XfNs: p<.003). N = neutral; F = fearful; f = face; s = scene; X = control (scrambled scene or triangle). (TIF) [file pone.0038118.s002.tif]
