# Supplementary material for: The Constructive Nature of Affective Vision: Seeing Fearful Scenes Activates Extrastriate Body Area
Source: PLoS One. 2012 Jun 29;7(6):e38118. doi: 10.1371/journal.pone.0038118 (PMC3387182; doi:10.1371/journal.pone.0038118)
Supplement: Table S1 — All individual Tal coordinates per subject. (DOC) [file pone.0038118.s003.doc]

| *Subject* | *x* | *y* | *z* | *voxels* |
| --- | --- | --- | --- | --- |
| 1 | 40 | -66 | 7 | 182 |
| 2 | 45 | -63 | 1 | 103 |
| 3 | 50 | -61 | 4 | 142 |
| 4 | 49 | -66 | 3 | 128 |
| 5 | 44 | -68 | 13 | 144 |
| 6 | 49 | -60 | 5 | 184 |
| 7 | 46 | -73 | 9 | 171 |
| 8 | 51 | -64 | 7 | 121 |
| 9 | 48 | -70 | 18 | 142 |
| 10 | 47 | -64 | 5 | 207 |
| 11 | 44 | -64 | -2 | 228 |
| 13 | 40 | -73 | 18 | 147 |
| 14 | 50 | -63 | 5 | 200 |
| 15 | 49 | -67 | 9 | 183 |
